# Psychosocial and behavioural characteristics in women with pregnancy-related lumbopelvic pain 12 years postpartum

**DOI:** 10.1186/s12998-019-0257-8

**Published:** 2019-08-13

**Authors:** Cecilia Bergström, Margareta Persson, Ingrid Mogren

**Affiliations:** 1 Department of Clinical Sciences, Obstetrics and Gynecology, Umeå University, 901 87 Umeå, Sweden; 2 Department of Nursing, Umeå University, 901 87 Umeå, Sweden

**Keywords:** Pregnancy-related lumbopelvic pain, Postpartum, Psychosocial characteristics, MPI, Cross-sectional questionnaire study

## Abstract

**Background:**

There is insufficient evidence regarding psychosocial factors and its long-term association with persistent pregnancy-related lumbopelvic pain. The overall aim of this study was to investigate women with persistent pregnancy-related lumbopelvic pain 12 years postpartum based on psychosocial and behavioural characteristics using the Swedish version of the Multidimensional Pain Inventory (MPI-S) classification system.

**Material and methods:**

This is a cross-sectional study based on a previous cohort. Data collection took place through a questionnaire. A total of 295 women from the initial cohort (*n* = 639) responded to the questionnaire giving a response rate of 47.3%. To determine the relative risk (RR) of reporting pain 12 years postpartum, a robust modified Poisson regression was used. This is the first study using the MPI-S as a predictive variable on women with persistent pregnancy-related lumbopelvic pain.

**Results:**

The MPI-S classification procedure was carried out on a total of *n* = 226 women, where 53 women were classified as interpersonally distressed (ID), 82 as dysfunctional (DYS), and 91 as adaptive copers (AC). Women in the ID and DYS subgroups had a relative risk (RR) of reporting persistent pregnancy-related lumbopelvic pain 12 years postpartum that was more than twice as high compared to the AC subgroup (95% confidence interval (CI) in parenthesis): RR 2.57 (CI 1.76 - 3.75), *p*<0.0001 and RR 2.23 (CI 1.53 - 3.25), *p*<0.0001 respectively. Women in the DYS subgroup had more than 5 times increased risk of reporting sick leave the past 12 months compared to the AC subgroup (RR 5.44; CI 1.70 - 17.38, *p*=0.004).

**Conclusions:**

The present study demonstrates that it is possible to classify women with persistent pregnancy-related lumbopelvic pain 12 years postpartum into relevant clinical subgroups based on psychosocial and behavioural characteristics using the MPI-S questionnaire.

## Introduction

Pregnancy-related lumbopelvic pain is not limited to pregnancy. Though most women recover within 6 months postpartum [1, 2], we as well as other researchers have demonstrated in several studies that symptoms can persist from a couple of years up to 12 years postpartum [3–8]. It has been shown that individuals with low back pain (LBP) who transition into a more chronic state take up the majority of the allocated resources [9]. The majority of the societal costs associated with chronicity are indirect cost such as disability, production loss, sickness absence and disability pension [10].

Psychosocial factors have long been associated with chronic pain and the bio-psycho-social model has become the leading theory of the development and management of chronic pain [11]. Psychosocial factors have also been demonstrated to play a crucial role in the transition from acute and sub-acute pain to chronicity [12–14]. In patients with musculoskeletal pain, psychosocial factors appear to exacerbate the clinical component of pain [15, 16] and have shown to influence future disability, pain, self-reported improvement after treatment in patients with LBP [17–21]. Even though pregnancy itself negatively influences health related quality of life lumbopelvic pain increases this influence [22]. Pregnancy-related lumbopelvic pain has also been shown to have great negative emotional and psychological impact on women [23]. Daily stress has been demonstrated to be a risk factor for pregnancy-related lumbopelvic pain [24] and women with postpartum depressive symptom are three times more likely to report lumbopelvic pain compared to those without [25].

The West Haven Multidimensional Pain Inventory (MPI) [26] is commonly used in studies concerned with chronic pain and was designed to capture the multidimensionality of chronic pain. Three clinically relevant subgroups are derived from the MPI instrument [27]; Interpersonally Distressed (ID), Dysfunctional (DYS), and Adaptive Copers (AC). Individuals in the ID subgroup is characterised by inadequate social support, and individuals in the DYS subgroup is characterised by high disability, affective distress, and pain intensity, while the AC group demonstrates a more successful adjustment to chronic pain.

There is a paucity of evidence in regard to psychosocial factors and its long-term association with pregnancy-related lumbopelvic pain. Therefore, the overall aim of this study was to investigate women with persistent pregnancy-related lumbopelvic pain 12 years postpartum based on psychosocial and behavioural characteristics using the MPI-S classification system. More specifically, we wanted to determine if women classified as ID or DYS, were more likely to report pain 12 years postpartum compared to women in the AC subgroup. Secondly, we wanted to investigate if women assigned to the ID and DYS subgroup were more likely to report widespread pain, higher pain intensity, more days with pain, sick leave and disability pension compared to women in the AC subgroup. In addition, we also wanted to explore the use of prescription/non-prescription drugs and treatment sought among women with pregnancy-related lumbopelvic pain based on psychosocial and behavioural characteristics using the MPI-S. To the best of our knowledge the MPI has not been used for this condition previously.

## Methods

### Study design

This study is a cross-sectional study based on a previous cohort consisting of newly delivered women reporting lumbopelvic pain during their pregnancy in 2002. All women who had delivered from 1 January 2002 to 30 April 2002 at Departments of Obstetrics and Gynaecology at Umeå University Hospital (UUH), and Sunderby Hospital (SH), in northern Sweden were invited to fill out the first questionnaire (Q1).

### Data collection

Collection of data took place through four questionnaires; distributed right after delivery (Q1), 6 months after delivery (Q2), 14 months postpartum (Q3), and 12 years postpartum (Q4). To enable comparisons over time, similar questions were posed throughout the questionnaires. Additionally, instruments that have been shown to work well with patients with chronic musculoskeletal pain such as the EQ-5D [28, 29], Roland Morris Disability Questionnaire (RMDQ) [30], and the Swedish version of the Multidimensional Pain Inventory (MPI-S) [31], were included in Q4. Data collection of Q4 took place between May and June 2014. More detailed information regarding the data collection has been presented in previous publications [6, 32].

### Study participants

**Fig. 1:**
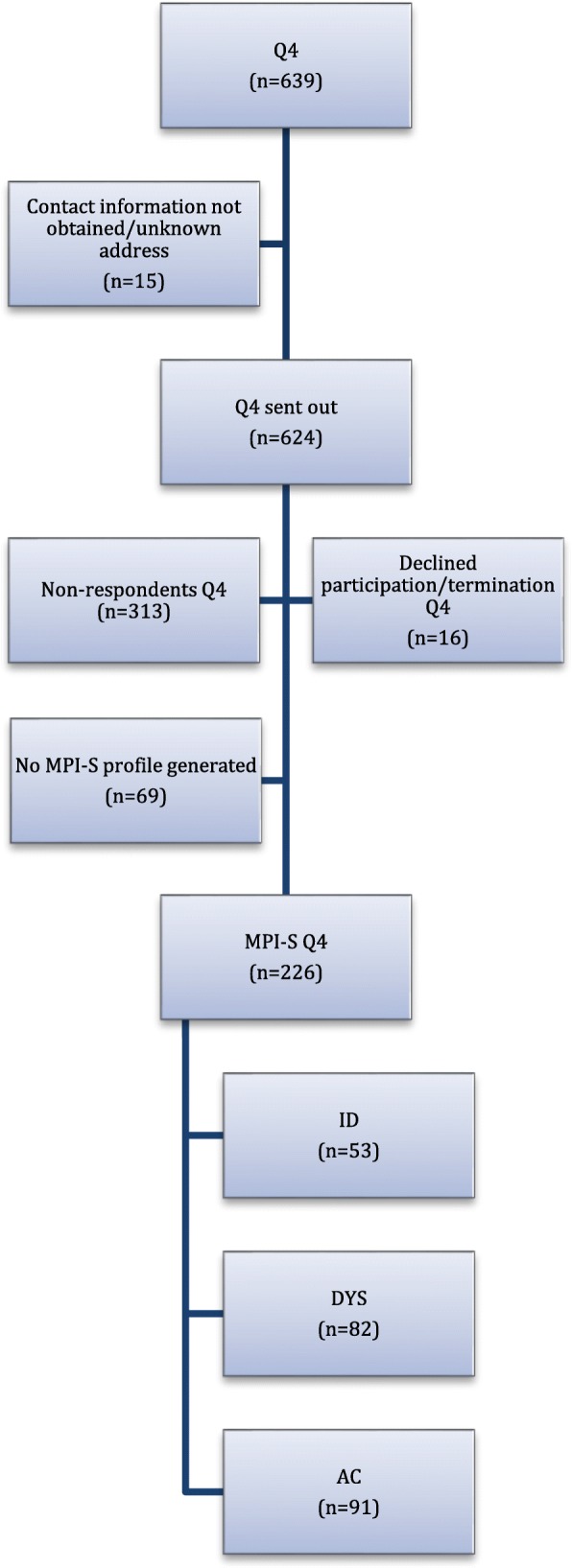
Flowchart of the study

The fourth questionnaires (Q4) were sent out to 624 women from the initial cohort (*n* = 639). A total of *n* = 295 women responded to the questionnaire giving response rate of 47.3% (Fig. 1).

### Dependent variables

The primary outcome measure was self-reported persistent pregnancy-related lumbopelvic pain 12 years postpartum using the previous definition in questionnaire Q1-Q3, where persistent pregnancy-related lumbopelvic pain was defined as ‘continuous’ or ‘recurrent’ pain in the lumbopelvic area over the past 12 months. The response alternative were ‘yes, continuous pain’, ‘yes, recurrent pain’, ‘yes, pain on a few occasions’, and ‘no pain’. Women reporting ‘continuous’ or ‘recurrent’ pain the past 12 months were also asked to mark the area of pain on a schematic pain drawing [32]. The outcome variable was dichotomized into ‘pain’ and ‘no pain’ where women reporting pain on a few occasions were considered not to be in pain at Q4.

As secondary outcomes, neck pain (NP) and/or thoracic spinal pain (TSP) were dichotomized the same way as pain in the lumbopelvic area. Sciatica was defined as pain in one or both legs in relation to lumbopelvic pain the past 12 months. Pain intensity the past week and the past 12 months was reported through a visual analogue scale (VAS) of 100 mm (mm), where 0 indicated ‘no pain’ and 100 ‘worst imaginable pain’. Pain intensity was dichotomized into scores above and below 70 mm on the VAS where women scoring ≥70 mm were considered to be in severe pain [33, 34]. This cut-off score has previously been used for the same study group [4, 32, 35]. The participants were asked to estimate how many days the past 12 months they have had lumbopelvic pain with the response alternatives ‘less than 30 days’ and ‘more than 30 days’. The same classification was done for days with NP/TSP the past 12 months. Participants were asked if they had been on sick leave due to lumbopelvic pain the past 12 months (response alternative ‘yes’ or ‘no’) and if so, how many days with the options ‘1-7 days in total’, ‘8-14 in total’, and ‘more than 15 days in total’ (dichotomized to < or ≥ 15 days the past 12 months), and to what degree they had been on sick leave (‘full-time’ and ‘part-time’ including to what degree in percentage). To investigate granted disability pension, the question ‘have you been granted disability pension due to lumbopelvic pain’ was used with response alternatives ‘yes’ and ‘no’.

To explore the use of prescription/non-prescription drugs the question ‘do you take any prescription and/or non-prescription drugs on a regular basis’ was used with the response alternatives ‘yes’ or ‘no’. The participants were also asked to write the names of the prescription/non-prescription drug/drugs they took on a regular basis. Furthermore, participants were asked if they had sought any healthcare/treatment due to lumbopelvic pain after their last pregnancy with the response alternatives ‘yes’ or ‘no’.

### Independent variables

Data regarding the participants’ psychological and behavioural profile were collected at Q4 through the MPI-S instrument. The MPI is a psychometric tool developed to categorize chronic pain patients into more homogenous subgroups of patients [26, 27]. The MPI has been used in a variety of chronic pain condition such as NP and LBP [27, 36–39], headaches [40], temperomandibular disorders [41], fibromyalgia [42], and cancer pain [43]. The MPI classification strategy has been shown to be independent of age, pain duration, pathology, and has been replicated in several studies [44].

The Swedish version of the MPI (MPI-S) has shown acceptable reliability and validity across gender [45]. The MPI-S is comprised of 34 items, 8-scale inventory divided into one psychosocial and one behavioural section. The psychosocial section consists of five scales: pain severity (PS), pain-related interference of everyday life (I), perceived life control (LC), affective distress (AD), and perceived support from significant other (S). The behavioural part entails three scales measuring individual’s perception of responses of significant others to display of pain and suffering. The three scales are: punishing responses (PR), solicitous responses (SR), and distracting responses (DR). All scales include a 7 numerical interval between 0 and 6, where high scores indicate more of the characteristic in question.

Three different groups have empirically been derived from the MPI scales through cluster analysis and have been labelled: interpersonally distressed (ID), dysfunctional (DYS), and adaptive copers (AC) [27]. Individuals in the ID subgroup are characterised by inadequate social support, low solicitous response from significant other, and lower distracting response while individual in the DYS subgroup is characterised by high disability, affective distress, and pain intensity. Furthermore, individuals in the DYS subgroup are found to be more depressed, have more catastrophizing thoughts, low physical functioning, poor sleep quality and poor lifting capacity. The AC subgroup consists of individuals reporting a more successful adjustment to chronic pain compared both to ID and DYS individuals. AC individuals generally report low pain severity, low emotional distress, less catastrophizing thoughts, and better quality of sleep and physical functioning. The AC subgroup is considered to have the most favourable prognosis and less reported sick leave than both ID and DYS [37].

As not all responders had dated their questionnaire, mean age was calculated by subtracting the date of birth from January 1, 2015. Physical activity was investigated by asking the participants if they had exercised/done sports on a regular basis since their last pregnancy, with the response alternatives ‘yes’ or ‘no’. Body Mass Index (BMI) at Q4 was calculated by kilograms (kg)/height^2^ (meters). Self-rated health status (SRH) was investigated by asking the study participant to assess their current overall health status with the options: ‘very good’, ‘quite good’, ‘fair’, ‘quite poor’, and ‘poor’. The options for relationship satisfaction were: ‘very good’, ‘good’, ‘neither good or bad’, ‘bad’, and ‘very bad’.

### Statistical methods

The method used to derive the three different MPI-S subgroups was a non-hierarchical cluster procedure (K-Means algorithm). Computations started with a standardisation of the MPI-S scales using the mean value and standard deviation to form Z-scores and then T-scores. The MPI-S subgroups were formed from the eight original scales using centroid vectors from a previously validated sample [31].

Descriptive statistics were used for all background variables of the three different MPI-S groups through the calculation of means and standard deviation (SD) for parametric data using one-way ANOVA. To test for differences between the MPI-S subgroups the independent t-test and Pearson’s chi-square test was used as appropriate.

The dichotomized primary outcome of persistent pregnancy-related lumbopelvic pain 12 years postpartum was analysed using a robust modified Poisson regression [46] to determine the relative risk (RR) of reporting pain 12 years postpartum. The MPI-S subgroups were used as the predictive variable in the model. The same procedure was used for the secondary outcomes: pain intensity, days with pain, and sick leave as well as for the use of prescription/non-prescription drugs and treatment sought the past 12 months. The final model of the robust modified Poisson adjusted for LBP prior to pregnancy in 2002 improved the Akaike's Information Criterion (AIC) and the Bayesian Information Criterion (BIC). Due to violation of the normality assumption, pain intensity the past week as well as the past 12 months were also analysed by Kruskal-Wallis 1-way ANOVA on ranks; a suitable alternative for comparisons of three groups or more [47]. Additionally, the Mann-Whitney *U* test was used to compare difference between two independent variables. As individuals belonging to the AC subgroup are considered better copers, the AC subgroup was used as the predefined reference group in all statistical analyses. All data was analysed using the SPSS v 24.0 software package. Statistical significance was set at *p* < 0.05 when comparing differences among the three MPI-S subgroups.

### Ethics, consent and permission

This study was approved by the Ethics Committee at the Umeå University (Dnr 2014–4-32 M supplement to Dnr 2012–404-31 M) and was performed in accordance with the Declaration of Helsinki. Written informed consent was obtained from all participants. No collection of details, images, or videos related to an individual person took place in this study.

## Results

### The Swedish version of the MPI (MPI-S)

**Fig. 2:**
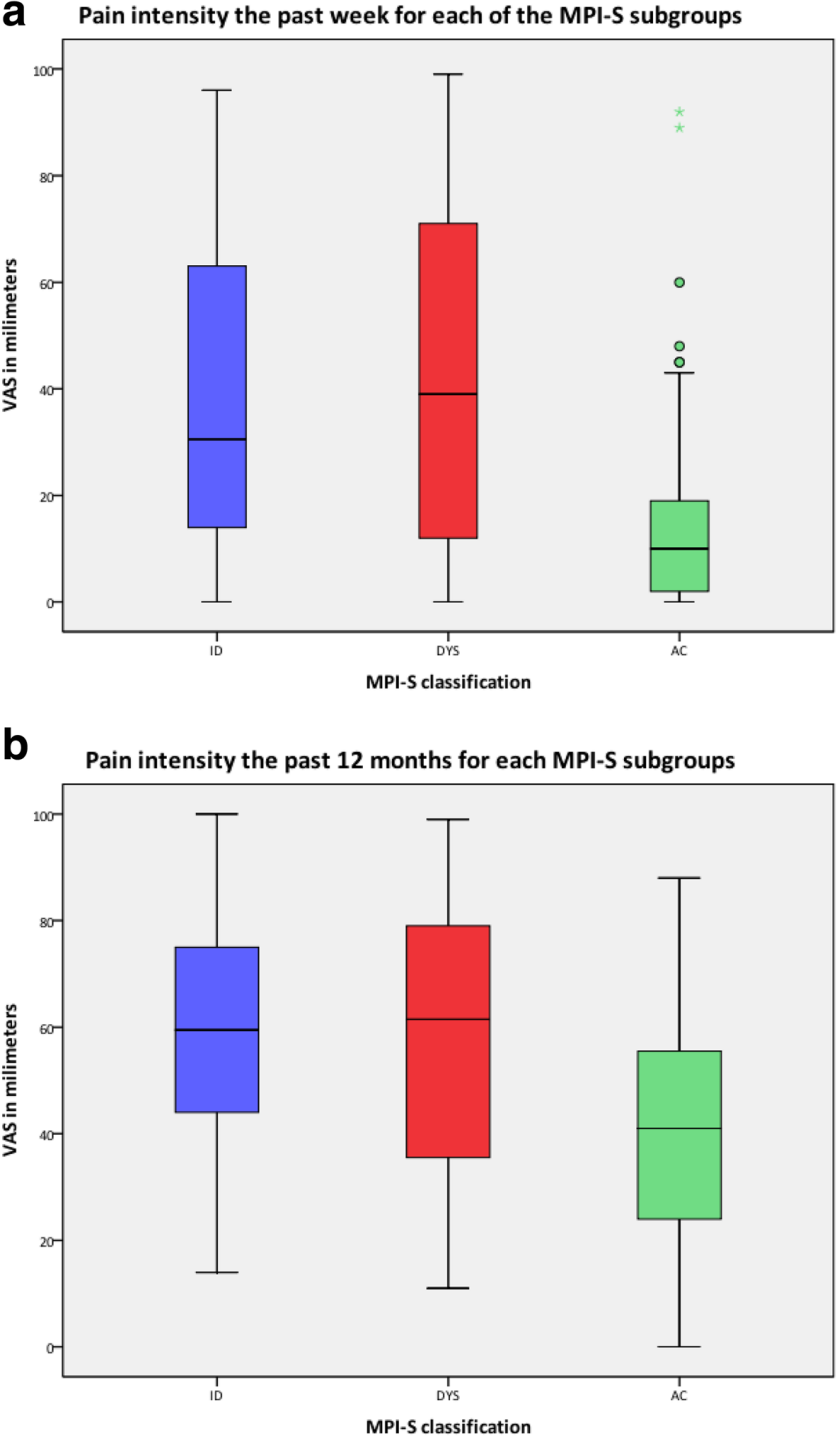
**a** Pain intensity the past week for each of the MPI-S subgroups. **b** Pain intensity the past 12 months for each of the MPI-S subgroups

The MPI-S classification procedure was carried on a total of *n* = 226 women. Table 1 describes the MPI-S study population at Q4, where 53 women were classified as interpersonally distressed (ID), 82 as dysfunctional (DYS), and 91 as adaptive copers (AC). The mean age across the MPI-S group was 43.4 years (SD 4.7). The vast majority of women were either married or cohabiting and 53.6% had a high school/folk school education. Most women reported having a total of 2 or 3 children and almost 70% of the women reported to participate in physical activity on a regular basis. Mean BMI across the study population was 25.2 (SD 4.4) kg/m^2^ and most women reported to be satisfied with their relationship. No statistically significant difference was found between the MPI-S subgroups regarding the above-mentioned variables in Table 1. Women in both the ID and DYS subgroup reported LBP before pregnancy in 2002 to a statistically significantly higher degree compared to the AC subgroup (*p* = 0.025). Additionally, a statistically significant difference were found between women in the ID and DYS subgroups compared to the AC subgroup in regard to pain status (pain versus no pain) at Q4 (*p* < 0.0001), pain intensity the past week (*p* < 0.0001) and the past 12 months (*p* < 0.0001), and SRH (*p* < 0.0001), where women in the AC subgroup reported pain at Q4 to a lesser degree, lower pain intensity both the past week (Fig. 2a) as well as the past 12 months (Fig. 2b). Women in the AC subgroup also reported better SRH at Q4 compared both to both the ID and DYS subgroups.

**Table 1:** Descriptive information on MPI-S subgroups

	MPI-S subgroups			Total *n* = 226	*P*-value^e^	
	ID^a^ *n* = 53	DYS^b^ *n* = 82	AC^c^ *n* = 91		*X* ^2^	*F*
Age, mean (SD)	43.8 (5.1)	42.8 (4.5)	43.6 (4.6)	43.4 (4.7)		0.38
Marital status						
Married/cohabiting	42 (85.7)	65 (92.2)	65 (91.5)	172 (90.5)	0.27	
Relationship but not cohabiting	2 (4.1)	4 (5.7)	2 (2.8)	8 (4.2)		
Single	5 (10.2)	1 (1.4)	4 (5.6)	10 (5.3)		
Education in 2002						
Up to high school/folk school	29 (54.7)	43 (53.8)	47 (52.8)	119 (53.6)	0.96	
University or higher	24 (45.3)	37 (46.3)	42 (47.2)	103 (46.4)		
Total number of children						
1	14 (26.4)	16 (19.5)	23 (25.3)	53 (27.0)	0.72	
2	16 (30.2)	21 (25.6)	28 (30.8)	65 (33.2)		
3	12 (22.6)	26 (31.7)	26 (28.6)	64 (32.7)		
≥ 4	11 (20.8)	19 (23.2)	14 (15.4)	44 (22.4)		
Physical activity						
Yes	33 (62.3)	53 (66.3)	66 (73.3)	152 (68.2)	0.35	
No	20 (37.7)	27 (33.8)	24 (26.7)	71 (31.8)		
Body Mass Index (BMI), mean (SD)	25.0 (5.3)	24.9 (4.3)	25.9 (4.1)	25.2 (4.4)		0.67
Relationship satisfaction						
Very good	18 (37.5)	47 (58)	54 (62.8)	119 (55.3)	0.13	
Good	18 (37.5)	23 (28.4)	24 (27.9)	65 (30.2)		
Neither good or bad	9 (18.8)	9 (11)	7 (8.1)	25 (11.6)		
Bad	2 (4.2)	2 (2.4)	1 (1.2)	5 (2.3)		
Very bad	1 (2.1)	–	–	1 (0.5)		
Low back pain before pregnancy in 2002						
No	5 (27.8)	21 (48.8)	18 (69.2)	44 (50.6)	0.025	
Yes	13 (72.2)	22 (51.2)	8 (30.8)	43 (49.2)		
Pain status						
No pain	14 (26.9)	30 (36.6)	65 (72.2)	109 (48.7)	< 0.0001	
Pain	38 (73.1)	52 (63.4)	25 (27.8)	115 (51.3)		
Pain intensity the past week (VAS^d^), mean (SD)	38.0 (28.6)	41.4 (31.9)	15.2 (19.7)	31.68 (29.8)		< 0.0001
Pain intensity the past 12 months (VAS^d^), mean (SD)	58.7 (23.4)	52.2 (23.9)	40.4 (20.0)	52.3 (23.9)		< 0.0001
Self-reported health (SRH)						
Very good	3 (5.7)	2 (2.5)	24 (26.4)	29 (12.9)	< 0.0001	
Quite good	19 (35.8)	29 (35.8)	52 (57.1)	100 (44.4)		
Fair	18 (34.0)	28 (34.6)	13 (14.3)	59 (26.2)		
Quite poor	12 (22.6)	17 (21.0)	1 (1.1)	30 (13.3)		
Poor	1 (1.9)	5 (6.2)	1 (1.1)	7 (3.1)		

Numbers in parenthesis are percentage unless otherwise specified

^a^Interpersonally distressed

^b^Dysfunctional

^c^Adapative copers

^d^Visual Analogue Scale

^e^Significance test *p* < 0.05

Table 2 gives an overview of descriptive data for the three individual subgroups (ID, DYS and AC). As expected, the descriptive data could differentiate between the individual subgroups and statistically significant differences were observed in all variables with the exception of NP/TSP the past 12 months, degree of sick leave and days of sick leave the past 12 months.

**Table 2:** Test for difference between MPI-S groups 12 years postpartum using Pearson’s chi-square test

	ID^a^ *n* = 53	DYS^b^ *n* = 82	AC^c^ *n* = 91	*p*-value^e^
Pain intensity the past week (VAS^d^)				
< 70 mm	43 (81.1)	62 (75.6)	89 (97.8)	< 0.0001
≥ 70 mm	10 (18.9)	20 (24.4)	2 (2.2)	
Pain intensity the past 12 months (VAS^d^)				
< 70 mm	37 (69.8)	55 (67.1)	88 (96.7)	< 0.0001
≥ 70 mm	16 (30.2)	27 (32.9)	3 (3.3)	
Days with persistent LBP/PGP the past 12 months				
< 30 days	11 (23.9)	24 (31.6)	45 (71.4)	< 0.0001
≥ 30 days	35 (76.1)	52 (68.4)	18 (28.6)	
Sciatica				
No	13 (28.3)	22 (29.3)	37 (58.7)	< 0.0001
Yes	33 (71.7)	53 (70.7)	26 (41.3)	
Neck or thoracic pain the past 12 months				
No	16 (51.6)	26 (59.1)	21 (77.8)	0.11
Yes	15 (48.4)	18 (40.9)	6 (22.2)	
Days with neck or thoracic pain the past 12 months				
< 30 days	11 (26.2)	21 (32.3)	25 (53.2)	0.018
≥ 30 days	31 (73.8)	44 (67.7)	22 (46.8)	
Sick leave the past 12 months				
No	41 (91.1)	56 (75.7)	58 (95.1)	0.003
Yes	4 (8.9)	18 (24.3)	3 (4.9)	
Degree of sick leave				
Full-time	6 (100)	15 (88.2)	2 (66.7)	0.34
Part-time	–	2 (11.8)	1 (33.3)	
Days of sick leave the past 12 months				
< 15 days	2 (50.0)	8 (44.4)	2 (50.0)	0.97
≥ 15 days	2 (50.0)	10 (55.6)	2 (50.0)	
Granted disability pension				
No	39 (88.6)	68 (90.7)	57 (100)	0.042
Yes	5 (11.4)	7 (9.3)	–	
Prescription and/or non-prescription drugs				
No	22 (41.5)	33 (40.7)	58 (65.2)	0.002
Yes	31 (58.5)	48 (59.3)	31 (34.8)	
Treatment sought since last delivery due to persistent LBP/PGP				
No	26 (56.5)	26 (34.2)	43 (70.5)	< 0.0001
Yes	20 (43.5)	50 (65.8)	18 (29.5)	

Numbers in parenthesis are percentage unless otherwise specified

^a^Interpersonally distressed

^b^Dysfunctional

^c^Adapative copers

^d^Visual Analogue Scale

^e^Significance test *p* < 0.05

### Primary and secondary outcomes

**Fig. 3:**
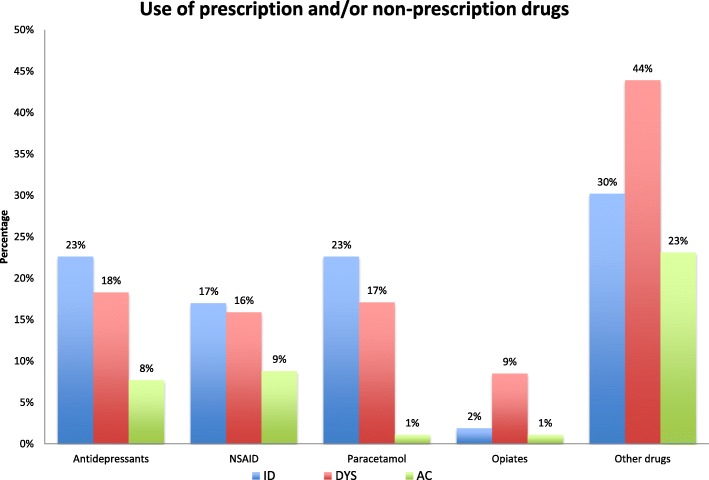
Use of prescription and/or non-prescription drugs for each of the MPI-S subgroups

Women belonging to the ID and DYS subgroup had a RR of reporting pain 12 years postpartum (95% confidence interval (CI) in parenthesis) of 2.57 (CI 1.76–3.75), *p* < 0.0001 and 2.23 (CI 1.53–3.25), *p* < 0.0001 respectively, compared to the AC subgroup (adjusting for LBP before pregnancy in 2002). Moreover, additional analyses demonstrated that women in the ID subgroup had a statistically significant increased RR in all variables with the exception of NP/TSP, sick leave and treatment sought the past 12 months compared to the AC subgroup. The DYS subgroups had a statistically significant increased RR in all variables, except NP/TSP, compared to the AC subgroup. Notable is that the DYS subgroup had more than 5 times increased RR of reporting sick leave the past 12 months compared to the AC subgroup at Q4 (RR 5.44; CI 1.70–17.38, *p* = 0.004) (Table 3). In addition, both the ID and DYS subgroup had an 8 to 11 times increased RR in reporting pain intensity of ≥70 mm both the past week and the past 12 months. Women in the ID and DYS subgroups also used antidepressants (χ^2^ (2, *N* = 226) = 6.92, *p* = 0.031), paracetamol (χ^2^ (2, *N* = 226) = 17.99, *p* > 0.0001), opiates (χ^2^ (2, *N* = 226) = 7.04, *p* = 0.03), and other drugs (χ^2^ (2, *N* = 226) = 8.70, *p* = 0.031) to a statistically significant higher extent compared to the AC subgroup with the exception of Non-Steroid Anti-Inflammatory Drugs (NSAID) (χ^2^ (2, *N* = 226) = 2.70, *p* = 0.26) (Fig. 3).

**Table 3:** Risk ratios for the MPI-S groups using AC^c^ as reference of the explantory variable, estimated by modified Poisson regression and adjusted for low back pain prior to pregnancy in 2002

	ID^a^				DYS^b^			
	n (%)	RR	CI (95%)	*p*-value^e^	n (%)	RR	CI (95%)	*p*-value^e^
Pain status 12 years post partum	49 (22.5)				79 (36.2)			
No pain		1				1		
Pain		2.57	1.76–3.75	< 0.0001		2.23	1.53–3.25	< 0.0001
Days with lumbopelvic pain the past 12 months	43 (24.2)				73 (41.0)			
<30 days		1				1		
≥30 days		2.63	1.72–4.03	< 0.0001		2.35	1.55–3.57	< 0.0001
Pain intensity the past week (VAS^d^)	50 (22.8)				79 (36.1)			
<70 mm		1				1		
≥70 mm		8.38	1.90–37.00	0.005		11.00	2.66–45.48	0.001
Pain intensity the past 12 months (VAS^d^)	50 (22.8)				79 (36.1)			
<70 mm		1				1		
≥70 mm		9.43	2.89–30.72	< 0.0001		9.32	2.94–29.57	< 0.0001
Sciatica	43 (24.3)				72 (40.7)			
No		1				1		
Yes		1.81	1.27–2.58	0.001		1.77	1.26–2.48	0.001
Neck and/or thoracic pain	28 (29.5)				41 (43.2)			
No		1				1		
Yes		2.09	0.94–4.62	0.07		1.98	0.92–4.26	0.08
Days with neck or thoracic pain the past 12 months	39 (26.5)				62 (42.2)			
<30 days		1				1		
≥30 days		1.62	1.12–2.34	0.01		1.51	1.05–2.16	0.03
Sick leave the past 12 months	42 (24.3)				71 (41.0)			
No		1				1		
Yes		2.13	0.51–8.82	0.30		5.44	1.70–17.38	0.004
Treatment sought the past 12 months	43 (24.4)				73 (41.5)			
No		1				1		
Yes		1.51	0.91–2.52	0.11		2.28	1.50–3.46	< 0.0001
Prescription and/or non-prescription drugs	50 (23.1)				78 (36.1)			
No		1				1		
Yes		1.66	1.13–2.43	0.009		1.70	1.20–2.41	0.003

Numbers in parenthesis are percentage unless otherwise specified

^a^Interpersonally distressed

^b^Dysfunctional

^c^Adapative copers

^d^Visual Analogue Scale

^e^Significance test *p* < 0.05

Table 4 show that there was a statistically significant effect on the overall pain intensity the past week as well as pain intensity the past 12 months between the MPI-S subgroups (*p* < 0.0001). A statistically significant difference was also demonstrated regarding pain intensity the past week and the past 12 months between ID and AC and between DYS and AC, where women in the ID and DYS subgroup reported higher pain intensity in both variables compared to the AC subgroup (Table 4).

**Table 4:** Comparing highest level of pain at Q4 using the AC group as the reference group

Highest level of pain	ID				DYS				*p*-value^c^ KW-test
	n	Median^a^	IR^b^	*p*-value MW-test	n	Median^a^	IR^b^	*p*-value MW-test	
Past week	46	30.5	51	< 0.0001	75	39	59	< 0.0001	< 0.0001
Past 12 months	46	59.5	34	< 0.0001	76	61.5	44	< 0.0001	< 0.0001

^a^Median Mann-Whitney U test. ^b^*IR* Interquartile Range. ^c^Kruskal-Wallis *p*-value

Significant result *p* < 0.05 using Mann-Whitney *U* test

Significant result *p* < 0.05 using Kruskal-Wallis test

## Discussion

This study aimed to investigate psychosocial and behavioural characteristics in women with persistent pregnancy-related lumbopelvic pain 12 years postpartum using the MPI-S. In this study we have demonstrated that it is possible to classify this group of women into clinically relevant subgroups using the MPI-S questionnaire. As hypothesized, women with more pronounced psychosocial difficulties (ID or DYS) were more than twice as likely to report pain 12 years postpartum compared to women belonging to the AC subgroup. Women in the ID and DYS subgroups also reported widespread pain to a higher extent, higher pain intensity, and more sick leave and disability pension compared to women in the AC subgroup. They also reported that they used prescription/non-prescriptions drugs to a higher degree and sought more treatment compared to women in the AC subgroup.

Together with previous episodes of LBP, psychological risk factors have been suggested to be of particular importance in the course of LBP [15, 16, 48–50]. Congruent with the findings in this study, emotional distress during pregnancy has been shown to be positively associated with severe persistent pregnancy-related lumbopelvic pain [51] and postpartum depressive symptoms has been demonstrated to be three times more prevalent in women with lumbopelvic pain compared to those not afflicted [25]. More extensive rehabilitation programs including counselling with focus on coping strategies may be more beneficial for patients with more pronounced psychosocial factors. A multidisciplinary approach is probably the most appropriate and effective intervention for patients with a variety of chronic musculoskeletal problem as it may help patients to lessens pain and disability, reduce number of sick days, and help the individual to faster return to work compared to physical treatment or usual care [52]. Consequently, it appears necessary to timeously address psychosocial factors in women with persistent pregnancy-related lumbopelvic pain in an attempt to prevent symptoms to become chronic in nature.

Poor health-related qualities of life, kinesiophobia, high degree of disability and pain intensity, have been demonstrated to be linked to pregnancy-related lumbopelvic pain [53]. Persistent symptoms seem to negatively affect the ability to perform daily activities and women with persistent symptoms postpartum are often concerned about the possible progression of symptom [54]. As expected, women classified as ID and DYS demonstrated statistically increased risk in almost all secondary outcome variables compared to the AC subgroup. It is noteworthy that there were more than 5 times increased risk of women in the DYS subgroup to report sick leave the past 12 months compared to the AC subgroup (Table 3). These findings are similar to results in previous studies using the MPI questionnaire on populations with chronic LBP [37, 38]. Another study found that individuals with high levels of fear-avoidance were twice as likely to believe that sick leave is a good treatment for LBP compared to individuals with low levels of fear-avoidance [55]. The individual beliefs about LBP, comorbidities, and coping abilities appear to be the most important reasons for sick leave due to LBP [56]. This could possibly explain why women belonging to the AC subgroup reported statistically significant less sick leave compared to the DYS subgroup, as the AC subgroup is characterized by less pain intensity and co-morbidities, better coping abilities, and have a more positive outlook on LBP.

Patients on analgesics medications incur higher cost to society [10] and the World Health Organisation (WHO) has ranked depression as the single largest contributor to global disability [57]. In this study, prescription/non-prescription drugs were used to a significant lesser extent in the AC subgroup compared to both the ID and DYS subgroup. Women in the ID subgroup reported use of antidepressants drugs to a statistically significant higher extent compared to women in the DYS and AC subgroup. Even though individuals belonging to the DYS subgroup have been found to be significantly more depressed compared to individuals in the ID subgroup [44], there may be different factors contributing to symptoms of depression in the two subgroups. Depressive symptoms in ID individuals may be more contributed to marital and interpersonally difficulties, lack of support from significant other compared to individuals in the DYS subgroup [31, 44]. ID individuals rated the quality of their interpersonal relationship as lower compared to both DYS and AC subgroups [44] and this was also true in this study, though not to a statistically significant degree. Worrisome is the high use of opiates among women in the DYS subgroup, especially considering the unknown long-term effectiveness and safety of opioids [58] as well as the emerging evidence that long-term opioid use increases the risk of abnormal menstruation and of menopausal symptoms in women [59].

### Methodological considerations

This study has some limitations that need to be discussed. Failure to respond to parts of the MPI questionnaire renders an unclassifiable profile [60, 61]. A total of 69 women did not respond to sufficient numbers of questions of the MPI-S questionnaire and had to be excluded due to missing data in the two different section of the MPI-S. This negatively impacted the number of women in each of the MPI-S subgroups, hence, reducing statistical power and increased the risk of Type II error in the analysis of the data. The MPI-S was only distributed at Q4 and may infer that the women in this study could have changed MPI-S groups over the course of the study. Even though classification changes can occur over time (mostly in the AC subgroup) several studies have confirmed the MPI classification system internal reliability, validity and generalizability both in interventional and observational studies in patients with chronic musculoskeletal pain [31, 62–64]. In addition, the Swedish version of the MPI (MPI-S) has shown good reliability and validity across gender [45] and no difference in pain duration or medical variables [31].

Conversely to studies where we investigated individuals with chronic musculoskeletal pain using the MPI-S [38, 65], the number of individuals in the AC subgroup is high. This seems reasonable, as approximately 60% of women responding to the Q4 reported no pain 12 years postpartum [6]. Moreover, studies investigating NP and LBP in a gainfully employed population have demonstrated that the AC subgroup is commonly larger than both the ID and the DYS subgroup [36, 37].

This cross-sectional study is part of a cohort of women that commenced in 2002. At that point, today’s definition of PGP based on positive diagnostic tests as well as pain upon palpation of the ligaments and joints of the pelvis was not available. Instead pain drawings were used in this study to describe pain location [6, 32]. However, it is impossible to exclude pain from the lumbar area or a combination of PGP and LBP, as it strongly correlates to the same anatomical location as non-specific LBP. Yet, it has been demonstrated that there is an increased risk of persistent pregnancy-related lumbopelvic pain if a woman is diagnosed with both LBP and PGP during pregnancy [66]. Swedish women under the age of 65 are overrepresented in the statistics regarding chronic LBP [67] and the estimated prevalence of LBP in women in the age group 40–49 years is 35% [68]. LBP is commonly regarded as stable over time, while pelvic pain increases during pregnancy [69]; hence factors and outcomes in this study are probably mostly related to pregnancy-related pelvic pain.

Baseline data (Q1) was complete on all subjects and analysis of the data collected through Q4 showed that non-responders did not differ significantly in the majority of variables compared to responders [6]. Convenience sampling of doublet questionnaires (Q4) were able to show that most questions showed adequate to excellent agreement [6] and questions in Q4 were very similar to those in Q1-Q3. Data collected after delivery (Q2-Q3) has been regarded to be representative of Swedish women with persistent PGP [32]. About 19% of women responding to Q4 reported pain to various degree 12 years postpartum, which is in line with other long-term follow-up studies of women with persistent pregnancy-related lumbopelvic pain [3, 8].

## Conclusions

This study is unique as it is the first study attempting to classify women with persistent pregnancy-related lumbopelvic pain into psychosocial derived subgroups. We were able to demonstrate that a multidimensional approach to classification of women with persistent pregnancy-related lumbopelvic pain based on psychosocial and behavioural characteristics, can further distinguish different clinically relevant subgroups in women with persistent symptoms 12 years postpartum. The MPI-S classification system together with clinical data, early and customized interventions with a multidisciplinary approach could thus improve clinical outcomes for women with persistent pregnancy-related lumbopelvic pain and reduce the economic burden on the social security and healthcare systems. Future research concerning women with pregnancy-related lumbopelvic pain needs to take psychosocial and behavioural characteristics into consideration.

## Abbreviations

AC: Adaptive copers
BMI: Body mass index
CI: Confidence interval
DYS: Dysfunctional
ID: Interpersonally distressed
LBP: Low back pain
MPI-S: Multidimensional Pain Inventory – Swedish version
NP: Neck pain
NSAID: Non-Steroid Anti-Inflammatory Drugs
PPGP: Persistent pelvic girdle pain
RMDQ: Roland Morris Disability Questionnaire
RR: relative risk
TSP: Thoracic spinal pain
VAS: Visual analogue scale
